# THE CLOSER THE BETTER? AGE DIFFERENCES IN EXPRESSING EMOTIONS TO OTHERS

**DOI:** 10.1093/geroni/igae098.3738

**Published:** 2024-12-31

**Authors:** Jane Stephenson, Enna Chen, Kathrine Whitman, Laura Carstensen

**Affiliations:** Stanford University, Stanford, California, United States; Stanford University, Menlo Park, California, United States; Stanford University, Palo Alto, California, United States; Stanford University, Stanford University, California, United States

## Abstract

Socioemotional Selectivity Theory posits that as people age and future time horizons shorten, they prioritize emotional meaning over exploration. As a result, they selectively narrow their social networks to focus on close and emotionally meaningful relationships. Thus, older adults may be more likely to express their feelings to close social partners and benefit more emotionally from sharing their feelings in close relationships as compared to more distant ones. In an experience sampling study collected at five randomly selected times per day for seven days, 180 participants (aged 18 to 93, M = 57.3) reported whether they talked to someone about their emotions, their relationship to that person (spouse/partner, family, friend, acquaintance, coworker, stranger), and whether they felt better after talking to that person. On average, participants felt better after talking to someone about their emotions regardless of their relationship with that person, although they felt significantly better when they shared with close social partners (spouse/partner, family, friend) as compared to peripheral social partners (acquaintances, coworkers, strangers). While age was not significantly associated with the likelihood of expressing feelings to another person, sharing feelings with a family member was associated with feeling significantly better among older participants as compared to younger ones. These findings indicate that sharing feelings with others, no matter their relationship, can benefit emotional well-being at all ages. However, close social relationships provide the most emotional benefit and familial ones appear to play an especially important role at relatively advanced ages.

